# Inflammatory Heart Disease in Multisystem Inflammatory Syndrome

**DOI:** 10.1007/s11886-024-02173-9

**Published:** 2025-01-08

**Authors:** Giulia La Vecchia, Marco Giuseppe Del Buono, Aldo Bonaventura, Alessandra Vecchiè, Francesco Moroni, Tommaso Sanna, Antonio Abbate

**Affiliations:** 1 Department of Cardiovascular and Pulmonary Sciences, Catholic University of the Sacred Heart, 00128 Rome, Italy; 2 Center of Excellence in Cardiovascular Sciences, Isola Tiberina Hospital Gemelli Isola, Rome, Italy; 3 Department of Cardiovascular Sciences, Fondazione Policlinico Universitario A. Gemelli IRCCS, Largo Agostino Gemelli, 1, 00168 Rome, Italy; 4 Ospedale Di Circolo E Fondazione Macchi, DepartmentofInternalMedicine, S.C. Medicina Generale 1, Medical Center, ASSTSetteLaghi, Varese, Italy; 5 Robert M. Berne Cardiovascular Research Center, and Department of Medicine, DivisionofCardiovascularMedicine,HeartandVascularCenter, University of Virginia, Charlottesville, VA USA

**Keywords:** Inflammation, Severe Acute Respiratory Syndrome Coronavirus 2, Pericarditis, Myocarditis, Heart failure

## Abstract

**Purposeof the Review:**

In this review article, we aim to provide an overview of the pathophysiology, the clinical features, the therapeutic management and prognosis of patients affected by Multisystemic inflammatory syndrome (MIS) with cardiac involvement, focusing on myocarditis and pericarditis.

**Recent Findings:**

MIS is a multiorgan hyperinflammatory condition due to a cytokine storm following (within 4–12 weeks) SARS-CoV-2 (Severe Acute Respiratory Syndrome Coronavirus 2) infection. First described in children, it also affects young adults without comorbidities, predominantly males with highly heterogeneous clinical manifestations, including cardiac involvement.

**Summary:**

Pericardial and myocardial involvement are prevalent among patients affected by MIS leading to different clinical manifestations including myocarditis with arrhythmias, acute heart failure and cardiogenic shock that significantly affect the patient's prognosis. The heterogeneity of its clinical features and the significant overlap with other hyperinflammatory diseases make the diagnosis particularly challenging. Moreover, the evidence on the efficacy of pharmacological treatments targeting the hyperinflammatory response is scarce, as well as data on long-term prognosis.

## Introduction

**Fig. 1:**
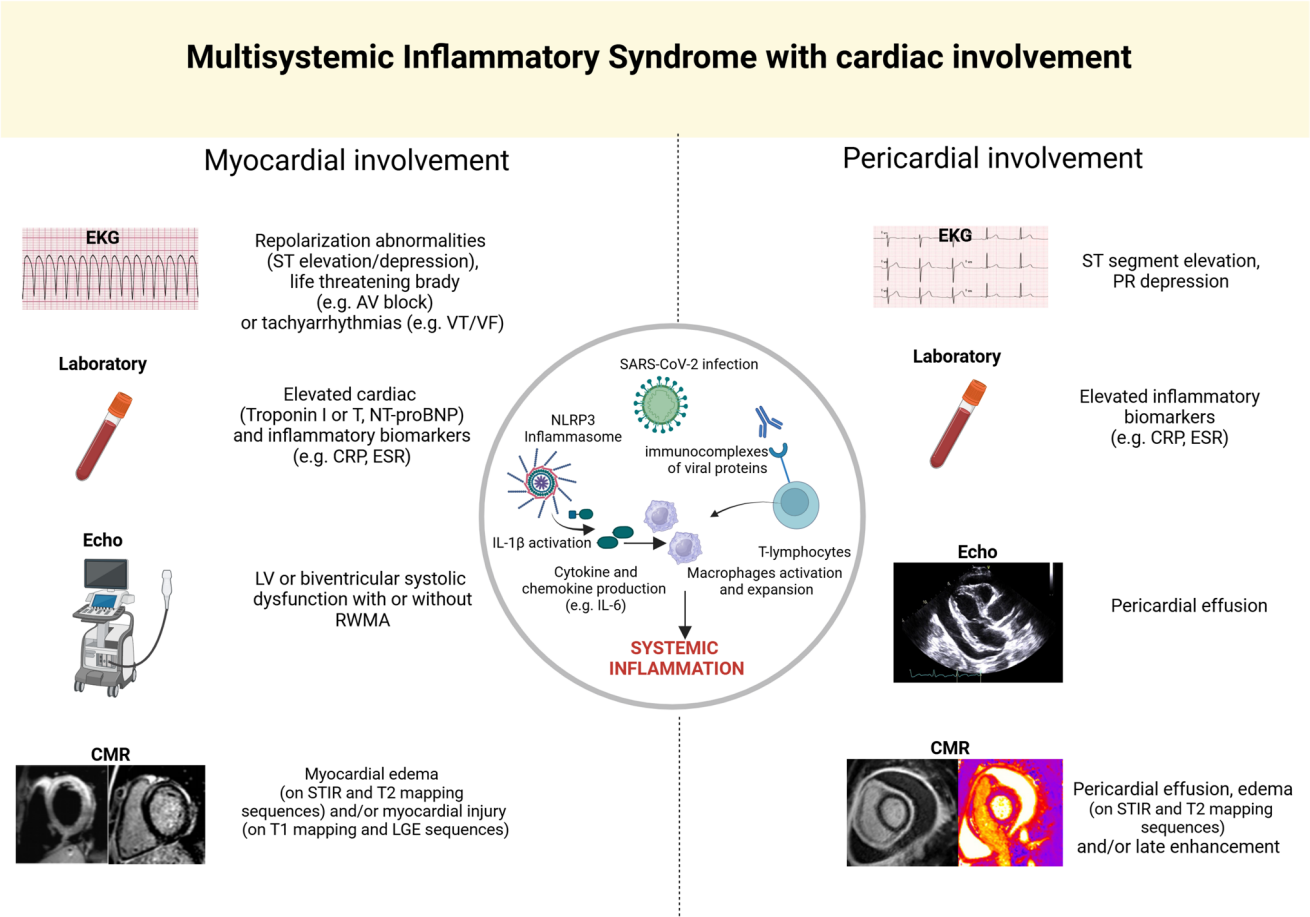
Myocardial and Pericardial Manifestations of Multisystemic Inflammatory Syndrome with Cardiac Involvement. The figure illustrates the clinical manifestations, laboratory and imaging (both at echocardiography and cardiovascular magnetic resonance) findings of Multisystem Inflammatory Syndrome (MIS) with myocardial and pericardial involvement. The figure summarizes the main pathophysiological mechanisms leading to the cytokine storm underlying the cardiac manifestations of this syndrome. Figure created with BioRender.com. *Abbrev*: AV: atrio-ventricular; ECG: electrocardiogram; CMR: cardiovascular magnetic resonance; CRP: C-reactive protein; echo: echocardiogram; ESR: erythrocyte sedimentation rate; IL: interleukin; LGE: late gadolinium enhancement; LV: left ventricular; NLRP3 inflammasome: pyrin domain-containing-3 inflammasome; NT-proBNP: N- terminal pro-b-type natriuretic peptide; RMWA: regional wall motion abnormality; SARS-CoV-2: severe acute respiratory syndrome coronavirus 2; STIR: short-tau inversion recovery; VES:; VT: ventricular tachycardia; VF: ventricular fibrillation

Multisystem Inflammatory Syndrome (MIS) is a rare but severe condition that emerged during the coronavirus disease 2019 (COVID-19) pandemic, primarily affecting children (MIS-C) and, to a lesser extent, adults (MIS-A) [1, 2]. It typically presents 4–12 weeks following severe acute respiratory syndrome coronavirus-2 (SARS-CoV-2) infection, and more rarely other bacterial or viral diseases, and is characterized by widespread systemic inflammation that can impact multiple organ systems [2, 3]. Among the most serious complications of MIS is cardiac involvement, particularly the inflammation of the myocardium (myocarditis) and the pericardium (pericarditis) with significant morbidity and, in some cases, mortality (Fig. 1) [2–17]. Despite the clinical relevance, the pathogenesis of cardiac involvement in MIS is poorly understood, and evidence supporting clinical management is scarce. This review article aims to provide an in-depth exploration of the myocardial and pericardial involvement in MIS, discussing the possible pathophysiology, clinical features, and the current knowledge gaps in the field.

### Epidemiology and Pathophysiology

The epidemiology of MIS is still unclear due to a lack of distinctive clinical features and easy-to-use definition. As such, the incidence, likely underestimated, remains relatively low compared to the total number of SARS-CoV-2 infections. In children, the highest incidence of this condition was documented among previously healthy individuals aged 5–14 years. A multicentre study by Feldstein and co-authors reported that patients diagnosed with MIS-C were more likely to be younger (mean age of 8.9 years, interquartile range [IQR] of 4.7 to 13.2 vs 11.7 IQR [1.2–16.6]) compared to patients with COVID-19, with a predominance of males affected (62% vs 38% of females) [17]. Moreover, MIS-C was more commonly observed among Black non-Hispanic and South Asian populations, though the reasons for this racial disparity are not fully understood [1, 17, 18]. Similarly, in the largest cohort of MIS-A by Patel et al. involving 221 subjects, patients affected were relatively young (median age 21 years), with no underlying comorbidity in most cases [5]. Moreover, males were predominantly affected (70% vs 30% of females) with a large prevalence of non-Hispanic Black individuals (36% of the overall population) as reported in children [5].

The pathophysiological mechanisms underlying MIS remain unknown, but it is believed to be a post-infectious immune-mediated condition. A hyperinflammatory state characterized by macrophage activation and elevated levels of pro-inflammatory cytokines, such as Interleukin-1β (IL-1β), IL-1α IL-6, and tumor necrosis factor (TNF) leads to a "cytokine storm" that in turn causes widespread tissue damage and amplifies the inflammatory response [2, 19]. In particular, the signaling of IL-1 family of cytokines plays a pivotal role in the pathogenesis of this syndrome. The IL-1 has two active forms: IL-1α and IL-1β.The latter is activated by inflammatory cells from its precursor, the pro-IL-1β, by caspase-1 enzymes which are triggered by the NLRP3 inflammasome [20–24].In MIS-C, the NLRP3 inflammasome pathway is probably activated by danger-associated molecular patterns (DAMPs), as elevated transcription products are observed during the acute phase of the disease [25]. Given the central role of NLRP3/IL-1β in cardiovascular diseases, it is likely that the NLRP3 inflammasome and IL-1β signalling also contribute to the cardiac involvement in MIS-A. The release of DAMPs and pathogen-associated molecular patterns (PAMPs) further activates innate immune cells, including macrophages and dendritic cells, leading to the secretion of additional pro-inflammatory cytokines like IL-6, which further amplify the inflammatory response and activate the adaptive immune response by stimulating T-cell development and antibody production to combat the viral infection [2, 26]. However, elevated IL-6 levels are also linked to immune dysregulation in pediatric illnesses, such as Kawasaki Disease (KD), as well as in COVID-19, where high IL-6 levels are associated with worse clinical outcomes [27, 28]. Thus, cytokines and ensuing activation of the adaptive immune response may in some cases trigger the immune-mediated processes further contributing to disease progression. Other molecular mechanisms involved in the pathogenesis of the disease include adaptative immune response dysregulation and T lymphocyte cell activation through epitope cross-reactivity with self-antigens and immune complexes formation [2]. Immunophenotyping of peripheral blood cells in patients with MIS-C showed characteristic changes in the proportions and functions of naïve CD4 + and CD8 + cytotoxic T cell counts, with significantly reduced levels of CD8 + , as were the ratios of monocyte and natural killer (NK) cell ratios [29, 30]. On the other hand, it has been documented a neutrophil and CD16 + monocyte increased activation and a high expression of migration proteins (ICAM-1), all indicating increased flow of myeloid cells to the periphery [31]. The reduced levels of circulating CD8 + and NK cells, together with enhanced activation of myeloid cells in the periphery contribute to maintaining the multiorgan systemic inflammation underlying MIS [29]. Cross-reactivity of viral epitopes with tissue-specific self-antigen or virus-encoded superantigens activating the CD4 + T lymphocytes have been also hypothesized as potential molecular pathways activated in cases of T-cell mediated myocarditis documented in adults [12]. Furthermore, circulating immunocomplexes of spike proteins of the virus have been isolated in patients affected by MIS-C, suggesting also a possible “superantigen-like response” where viral epitopes may elicit a non-specific polyclonal T cell activation and a massive cytokine release responsible for the hyperinflammatory state [32–34].

The reason for the higher prevalence of the disease among children is not completely understood. A stronger predisposition to an enhanced activation of the innate and adaptative immune response following the COVID-19 infection in children may lead to a more efficient clearance of the infection but a higher predisposition to develop MIS [5]. On the other hand, adults are more prone to delayed clearance of the virus due to immunosenescence and immune dysregulation, which can be responsible for more severe COVID-19-related symptoms rather than MIS. Furthermore, in older adults, the clinical presentation of MIS may be more complex due to coexisting autoinflammatory or severe comorbidities that make the diagnosis particularly challenging.

### Clinical Features and Differential Diagnosis of MIS

Extra-cardiac clinical features of MIS are remarkably heterogeneous and include persistent fever associated with signs of mucocutaneous involvement (annular macular rash or non-purulent conjunctivitis), gastrointestinal symptoms (vomiting, diarrhea), hematological (e.g. neutrophilia, but also lymphopenia and thrombocytopenia) and coagulation disorders (arterial and venous thrombosis). Moreover, patients with MIS typically present very high levels of circulating inflammatory biomarkers (C-reactive protein [CRP], IL-6, ferritin), generally higher compared to those found in patients with COVID-19 without MIS [17]. The above-mentioned clinical signatures are not exclusively associated with MIS but significantly overlap those of other pro-inflammatory conditions (e.g. Kawasaki disease [KD], Adult-Onset Still’s Disease [AOSD], the macrophage activation syndrome [MAS], viral myocarditis), thus making the diagnosis particularly challenging, especially in children (Table 1). Therefore, the Brighton Collaboration MIS-C Working Group proposed a case definition of MIS-C/A, which included the evidence of fever (subjective fever or documented fever [≥ 38.0 C]) and ≥ 2 clinical findings of systemic inflammation (mucocutaneous, gastrointestinal, neurological symptoms, hypotension/shock) (Table 2). Also, patients with MIS should have: (i) evidence of current or previous (within 12 weeks) SARS-CoV-2 infection; and (ii) signs of significant systemic inflammation and/or coagulopathy [1]. Signs and symptoms of cardiac involvement are considered under the primary diagnostic criteria, according to large population cohort studies reporting a predominant cardiovascular involvement among the distinctive signatures of the disease [5, 17]. These include evidence of myocardial and pericardial involvement (myocarditis or left ventricular systolic dysfunction [LVEF < 50%] and pericarditis), as well as coronary artery dilatation/aneurysm, more frequently observed in children, and brady- or tachi-arrhythmia (e.g. 2nd/3rd degree atrioventricular block, or ventricular tachycardia).

**Table 1:** Note: This data is Mandatory

	MIS	VIRAL MYOCARDITIS	KAWASAKI DISEASE	MAS	AOSD
Clinical features of systemic inflammation	Fever ± systemic vasodilation/ distributive shock	Fever over the previous 3–5 days	Fever ± systemic vasodilation /distributive shock (KDSS)	Non-remitting high fever	Daily recurring fever
Clinical manifestations of cardiac involvement	Myocarditis, pericarditis, coronary artery dilatation/ aneurysm, new‐onset LV/RV dysfunction (LVEF < 50%), tachy‐brady arrhythmias	New-onset LV/RV dysfunction (EF < 50%), pericarditis, tachy-brady arrhythmias	Coronary aneurysms, new-onset LV/RV dysfunction (LVEF < 50%) is less frequent	New onset LV/RV systolic dysfunction (LVEF < 50%), pericarditis	New onset LV/RV systolic dysfunction (EF < 50%), pericarditis
Extra-cardiac manifestations	Gastrointestinal symptoms, rash, non-purulent conjunctivitis, neurologic symptoms	Gastrointestinal symptoms pharyngodynia or symptoms of upper respiratory tract infections	Oral mucositis, conjunctivitis, rash, cervical adenopathy, hand and foot swelling, fingertip desquamation	Hepatosplenomegaly, lymphadenopathy, arthritis and disseminated intravascular coagulation	Arthritis, pharyngitis, evanescent and salmon-colored rash, lymphadenopathy
Laboratory	Lymphocytes < 1000 × 10^9^/L, platelet count < 150 × 10^9^/L, IL‐6 > 1.8 pg/mL, ferritin > 300 ng/mL (men) or > 150 ng/mL (women), fibrinogen > 400 mg/dL, NT‐proBNP > 125 ng/L, troponin I elevated, CRP > 10 mg/dL, procalcitonin < 0.05 ng/mL, erythrocyte sedimentation rate > 20 mm/h and positive COVID‐19 test via RT‐PCR, serology analysis, or antigen detection	Leukocytosis (> 11 × 109/L) with lymphocytosis (> 1000 × 109/L) or neutrophilia (> 80% or > 7500 × 109/L), CRP > 10 mg/dL, PCT > 0.05 ng/mL erythrocyte sedimentation rate > 20 mm/h raised Troponin I or T and BNP/NT-proBNP Positive RT-PCR, serology analysis and antigen detection	Leukocytosis(> 11 × 109/) with neutrophilia (> 80% or > 7500 × 109/L), platelet count > 500 × 109/L, CRP > 10 mg/dL, PCT < 0.05 ng/mL, erythrocyte sedimentation rate > 20 mm/h, fibrinogen > 400 mg/dL, elevated Troponin I or T and BNP/NT-proBNP	Ferritin level > 684 ng/mL plus any 2 of the following: pancytopenia (platelet count ≤ 181 × 10^9^/L, haemoglobin < 90 g/L, neutrophils < 1.0 × 10^9^/L), aspartate aminotransferase (AST) > 48 units/L, triglyceride concentration > 156 mg/dL, or fibrinogen ≤ 360 mg/dL	Leukocytosis (> 11 × 10^9^/L) with neutrophilia (> 80% or > 7500 × 10^9^/L), ferritin > 300 ng/mL (men) or > 150 ng/mL (women), CRP > 10 mg/dL, PCT < 0.05 ng/mL erythrocyte sedimentation rate > 20 mm/h
Cardiac imaging	Echo: LV/RV dysfunction with or without RWMA, MR, pericardial effusion CMR: LV/RV systolic dysfunction with or without RWMA with no coronary artery distribution, edema (on T2 weighted images) and/or myocardial injury with non-ischaemic distribution (LGE, raised native T1 mapping or ECV values), pericardial effusion with or without pericardial edema and/or late enhancement	Echo: LV/RV dysfunction with or without RWMA with no coronary artery distribution, pericardial effusion CMR: LV/RV systolic dysfunction with or without RWMA with no coronary artery distribution, edema (on T2 weighted images) and/or myocardial injury with non-ischaemic distribution (LGE, raised native T1 mapping or ECV values), pericardial effusion with or without pericardial edema and/or late enhancement	Echo: LV/RV wall motion abnormalities following the coronary artery distribution CMR: LV/RV systolic dysfunction with RWMA following the coronary artery distribution, edema (on T2 weighted images) and/or ischemic fibrosis (LGE), inducible myocardial ischaemia (by stress CMR)	Echo: LV/RV dysfunction with or without RWMA with no coronary artery distribution, pericardial effusion CMR: LV/RV systolic dysfunction with or without RWMA with no coronary artery distribution, edema (on T2 weighted images) and/or myocardial injury or fibrosis with non-ischaemic distribution (on LGE sequences) pericardial effusion with or without pericardial edema and/or late enhancement	Echo: LV/RV systolic dysfunction with or without RWMA with no coronary artery distribution, pericardial effusion

**Key:** *AOSD* Adult-onset Still’s Disease, *BNP* B-type natriuretic peptide, *CMR* Cardiovascular Magnetic Resonance, *CRP *C-reactive protein, *Echo* echocardiography, *ECV* extracellular volume, *EF* ejection fraction, *LGE* late gadolinium enhancement, *KDSS* Kawasaki Disease Shock Syndrome, *LV* left ventricular; MAS: Macrophage activation syndrome, *NT*-*proBNP* N-terminal pro b-type natriuretic peptide, *PCT* procalcitonin, *RT-PCR* reverse transcription-polymerase chain reaction, *RV* right ventricular, *RWMA* regional wall motion abnormality

(From: La Vecchia G, et al. J Am Heart Assoc. 2024 Feb 20;13(4):e032143, with permission from John Wiley and Sons) [2]

**Table 2:** Centers for Disease Control and Prevention Diagnostic Criteria of Multisystem Inflammatory Syndrome in Adults (MIS-A/C)

1. Documented fever (≥ 38 °C) for ≥ 24 h prior to hospitalization or within the first 3 days of hospitalization
2. Meet at least three clinical criteria (at least one must be a primary clinical criterion) within the first three days of hospitalization
a. *Primary clinical criteria*:
1. Severe cardiac illness: e.g. myocarditis, pericarditis, coronary artery dilatation/aneurysm, or new-onset right or left ventricular dysfunction (LVEF < 50%), 2nd/3rd degree A-V block, or ventricular tachycardia. Note: cardiac arrest alone does not meet this criterion, 2. Rash and non-purulent conjunctivitis
b. *Secondary clinical criteria*:
1. New-onset neurological symptoms (includes encephalopathy in a patient without prior cognitive impairment, seizures, meningeal signs, or peripheral neuropathy [including Guillain-Barré syndrome]
2. Shock or hypotension not attributable to medical therapy
3. Abdominal pain, vomiting, diarrhea
4. Thrombocytopenia (platelet count < 150 × 10^9^/L)
3. Meet laboratory evidence criteria
a. Elevated levels of at least two of the following: C-reactive protein, ferritin, interleukin-6, erythrocyte sedimentation rate, procalcitonin
b. A positive COVID-19 test via RT-PCR, serology, or antigen detection

**Key:** *A-V* atrio-ventricular, *COVID-19* Coronavirus Disease 2019, *LVEF* left ventricular ejection fraction, *RT-PCR* reverse transcription-polymerase chain reaction

(From: La Vecchia G, et al. J Am Heart Assoc. 2024 Feb 20;13(4):e032143, with permission from John Wiley and Sons) [2]

MIS can also mimic the clinical manifestations of a multi-organ involvement during the SARS-CoV-2 infection. However, compared to COVID-19 patients, individuals with MIS present more frequently with cardiovascular but not respiratory involvement, and with mucocutaneous manifestations of the disease [17]. Moreover, patients with MIS present more often neutrophilia and thrombocytopenia than lymphocytopenia and have higher levels of pro-inflammatory biomarkers (e.g. CRP and IL-6) within 48 h from admission compared to patients with COVID-19 [17].

### Cardiac Involvement in MIS

Cardiac involvement in MIS is common but heterogeneous and can range from mild myocardial dysfunction to severe, life-threatening conditions such as acute heart failure, cardiogenic shock (CS) and life-threatening arrhythmias. The cardiovascular system is more frequently involved in MIS-A than MIS-C, with a higher incidence of cardiac dysfunction documented in adults compared to children (54% vs 29%) [2, 16]. Moreover, in young adults it is described a higher rate of severe complications related to the cardiac involvement, with a larger proportion of deaths compared to what observed in patients with MIS-C (7% vs 1%) [5]. Among the cardiac manifestations of the disease, myocarditis and/or pericarditis are highly prevalent. A systematic review by Junior et al. reported a combined incidence of myopericarditis of 34% in patients with MIS-C [35]. Similarly, Patel et al. reported an incidence of myocarditis of 30% and of pericardial effusion of 25% in MIS-A patients [5]. Myocarditis may lead to various degrees of cardiac dysfunction [2–14]. Patients affected usually present with high levels of cardiac biomarkers (Troponin I or T, brain natriuretic peptide [BNP] or N-terminal pro B-type natriuretic peptide [NT-proBNP]), electrocardiographic (EKG) changes consistent with myocarditis (abnormal ST segments or low voltage QRS in the presence of edema), ischemia (abnormal ST segments, T wave inversion) or ventricular tachyarrhythmia (e.g. ventricular tachycardia [VT] or ventricular fibrillation [VF]). Bradyarrhythmia (atrioventricular or intraventricular conduction delay) and supra-ventricular tachyarrhythmias (atrial fibrillation or atrial flutter) are less frequent, but still possible manifestations accompanying the myocardial involvement of MIS-A [2]. Cardiac imaging modalities, such as transthoracic echocardiography and cardiac magnetic resonance (CMR) are the cornerstone diagnostic techniques to demonstrate myocardial damage [2]. In particular, transthoracic echocardiography can identify the presence and severity of left ventricular or biventricular systolic dysfunction (also assessed through ventricular myocardial speckle strain imaging), with or without regional wall motion abnormalities as well as diastolic dysfunction or less frequent complications such as valvulopathies (e.g. mitral regurgitation) [2]. CMR can provide more accurate quantification of cardiac volumes and function and pivotal diagnostic information on myocardial damage through its ability of tissue characterization. Previous studies showed that evidence of myocarditis according to the updated Lake Louise criteria can be found in up to 35% of patients with MIS-C, including the evidence of T2-based markers for myocardial edema (on short-tau inversion recovery [STIR] or T2 mapping sequences) and a T1-based markers for associated myocardial injury (late gadolinium enhancement [LGE], increased native myocardial T1 mapping and/or extracellular volume [ECV] values) [36–38]. An abundance of data from case series supports these findings also in adults [5, 7–12]. The CARDOVID study is one of the largest CMR cohort studies which evaluated 111 patients with MIS-C and cardiac involvement by CMR, performed within 28 days from the hospital admission [36]. Of interest, the authors found that myocardial injury demonstrated by the presence of LGE was evident in up to 90%, while the presence of edema was identified in up to 55% of patients, thus highlighting the possible transient nature of the myocardial injury and the importance of prompt execution of CMR study in suggestive cases [36]. A small cohort study by Scarduelli and co-authors, evaluating 24 patients with MIS-C and cardiac involvement in whom CMR was performed early after the onset of the symptoms (median delay of 6 days [4.3–10.3]) confirmed these findings, documenting the evidence of myocardial edema in 60% of patients and of LGE in up to 25% of patients. Of interest, the authors found that LGE on CMR was not correlated with the severity of the LV systolic dysfunction but patients with LGE were more likely to have a prolonged hospital stay thus underscoring the potential prognostic significance of these specific CMR findings [38].

Pericarditis usually presents uncomplicated, with rare cases of cardiac tamponade reported in children [39, 40]. Histopathological findings from a patient admitted for tamponade and MIS-C revealed various inflammatory cell infiltration of the pericardium, including CD8 + or CD4 + T cells, macrophages and granulocytes, while SARS‐CoV‐2 was not detected in the pericardial tissue [41]. These data support the inflammatory nature of the pericardial involvement, with potentially relevant therapeutic implications. Patients with pericarditis may have typical ECG abnormalities (PR segment depression, diffuse ST-segment elevation) and raised biomarkers of systemic inflammation (e.g. CRP, IL-6). In these cases, transthoracic echocardiography is fundamental in identifying the presence and extent of pericardial effusion as well as its hemodynamic impact on cardiac chamber filling or in guiding the interventional management (e.g. percutaneous pericardiocentesis) [42]. CMR and cardiac tomography (CT) are second-line imaging modalities that can be evaluated in selected complicated cases (e.g. recurrent or restrictive pericarditis) [42].

### Treatment

Randomized clinical trials supporting specific therapeutic strategies in MIS are lacking, and current evidence is based mainly on case series and expert consensus recommendations. Given the pathogenesis of this hyperinflammatory syndrome, the pharmacological management includes immunomodulatory therapies aimed at targeting the immune dysregulation and the cytokine storm responsible for the multi-organ involvement in addition to hemodynamic and pharmacological supportive treatments [2]. IVIG is the first-line therapy, particularly in cases with cardiac involvement where they have been shown to improve myocardial dysfunction and limit the course of the illness [2, 7, 8, 11, 43]. IVIG can modulate the immune response by several mechanisms including neutralizing pathogenic autoantibodies and suppressing inflammatory cytokines [7, 8]. Consensus documents support the use of IVIG administered at a dose of 2 g/kg in one single or four divided infusions every 6 h in adult patients with documented cardiac dysfunction and in MIS-C cases within the first ten days from the disease onset [43]. Intravenous (IV) glucocorticoids are a cornerstone of MIS therapy and should always be considered alone or in combination with IVIG, especially in severe or refractory cases [2]. In particular, low-to-moderate-dose glucocorticoids (methylprednisolone intravenously [IV] or per os 0.3–0.4 mg/die initial dose tapered over time) may be used alongside IVIG in patients with CS or in those who have still not developed severe end-organ involvement but with other concerning features of disease severity (e.g. severe LV systolic dysfunction). In cases with fulminant myocarditis and refractory CS, high doses glucocorticoids (methylprednisolone IV up to 1 g IV for 3 days, followed by 1–2 mg/kg per os to be tapered over 8–12 weeks) should be considered [2, 7, 8, 10–12, 44]. When IVIG and glucocorticoids are insufficient or not tolerated, biological agents targeting specific cytokines can be employed. Anakinra (Kineret®) is an IL-1 receptor antagonist. The adult dose is 100 mg daily by subcutaneous injection but can be used at higher doses and IV, up to 10 mg/kg daily [45]. In children, the recommended dosage is 1-2 mg/kg per dose and up to 4–8 mg/kg per dose once daily in KD. Sparse data documented favorable outcomes in refractory cases of MIS-C/A treated with anakinra as adjunctive pharmacological treatment or as a corticosteroid-sparing strategy, with a good efficacy and safety profile [1, 2, 46–48].

In case of clinical worsening despite an adequate dose of anakinra within 24–48 h of treatment, other immunomodulatory agents can be considered. Tocilizumab targets selectively the IL-6 receptor, blocking the downstream activation of the IL-6 pathway. This leads to a reduced production of inflammatory cytokines, acute-phase proteins, and other mediators that contribute to inflammation and tissue damage [49]. To date, consistent data support the efficacy of tocilizumab in children after a single intravenous dose administration, while evidence in adults are still scarce [1, 2, 10, 50–52].

### Prognosis

Patients with MIS usually present a favourable prognosis with a complete resolution of cardiac abnormalities at follow-up**.** In subjects with myocardial involvement, a residual LV systolic dysfunction assessed by left ventricular ejection fraction (LVEF) has been described, ranging from 5 to 10% in different case series [1, 53]. However, a persistent subclinical myocardial dysfunction detectable by impaired values of myocardial strain (left ventricular global longitudinal strain [LVGLS] > -18%) at 3–6 months follow-up is documented. A prospective study by Sirico et al. including 32 patients reported abnormal LVGLS values at 6 months follow-up in 13% of MIS-C cases with cardiac involvement [37]. Similarly, Scardurelli and co-authors showed that, despite the improvement in terms of LVEF in all patients, LVGLS significantly differ among MIS vs non-MIS patients at 3 months follow-up [38]. The same authors showed also that subjects with evidence of myocardial injury by CMR at the time of admission (25%) had complete resolution at 6-month follow-up CMR, with no residual LGE [38]. Other studies reported the persistence of a non-ischemic LGE at 6-month follow-up in up to 30–35% of patients, despite a reduction in terms of extension of segments involved by quantitative analysis [37]. These data suggest that in most cases the LGE observed in the acute phase of the disease is due to myocardial injury rather than myocardial scar, with a favorable prognosis in terms of arrhythmic complications and heart failure development among most patients. Prognostic data about patients with pericarditis are scarce, and the rate of recurrence or long-term complications (e.g. constrictive pericarditis) is lacking to date.

## Conclusions

In conclusion, MIS-C/ A represents a complex, hyperinflammatory condition with significant cardiac implications, including myocarditis and pericarditis. The involvement of the myocardium and pericardium in MIS presents a spectrum of clinical challenges, from mild myocardial dysfunction to severe life-threatening conditions such as cardiogenic shock and ventricular arrhythmias. Despite advancements in understanding the pathophysiological mechanisms, including the pivotal role of cytokine storms and immune dysregulation, the heterogeneity of clinical presentations and the overlap with other inflammatory conditions complicate diagnosis and management. Current therapeutic approaches, largely based on immunomodulatory therapies, have shown promise in improving cardiac function and patient outcomes. However, the lack of randomized clinical trials underscores the need for further research to refine treatment protocols and improve long-term prognosis. Ultimately, while the prognosis for most patients is favorable, with resolution of cardiac abnormalities in the majority, ongoing surveillance is essential to identify and manage potential long-term complications, particularly in those with significant initial cardiac involvement.

## Key References

- La Vecchia G, Del Buono MG, Bonaventura A, Vecchiè A, Moroni F, Cartella I, Saponara G, Campbell MJ, Dagna L, Ammirati E, Sanna T, Abbate A. Cardiac Involvement in Patients With Multisystem Inflammatory Syndrome in Adults. J Am Heart Assoc. 2024 Feb 20;13(4):e032143.
  - This review article comprehensively summarizes the clinical features and therapeutic strategies of MIS with cardiac involvement in adults.
- Patel P, DeCuir J, Abrams J, Campbell AP, Godfred-Cato S, Belay ED. Clinical Characteristics of Multisystem Inflammatory Syndrome in Adults: A Systematic Review. JAMA Netw Open. 2021 Sep 1;4(9):e2126456.
  - Findings from this meta-analysis provide real-world data on the prevalence, clinical manifestations, and therapeutic management of patients with MIS and cardiac involvement.

## Data Availability

No datasets were generated or analysed during the current study.
